# Spontaneous Renal Artery Dissection in a Man with Previous Spontaneous Superior Mesenteric Artery Dissection

**DOI:** 10.1155/2020/4726381

**Published:** 2020-02-11

**Authors:** Yujiro Yokoyama, Masato Nakajima

**Affiliations:** ^1^General Surgery Department, Easton Hospital, 18042, USA; ^2^Cardiovascular Surgery Department, Yamanashi Prefectural Central Hospital, 400-8506, Japan

## Abstract

Both spontaneous superior mesenteric artery dissection (SMAD) and spontaneous renal artery dissection (SRAD) are very rare conditions. Their etiologies and natural histories are not precisely defined, but they are thought to be associated with underlying conditions. In this report, we describe an extremely rare case of SRAD in a man who had a history of spontaneous SMAD. We successfully treated SRAD with endovascular intervention. Isolated spontaneous SMAD and SRAD are both rare conditions. Their optimal treatment has not been established due to their rare entities, but endovascular treatment is a good option because it can prevent both advancement of infarction and renovascular hypertension, and it has become safer as device technology has improved. Patients with isolated visceral artery dissection should be carefully followed up.

## 1. Introduction

Both spontaneous superior mesenteric artery dissection (SMAD) and spontaneous renal artery dissection (SRAD) are very rare conditions. Their etiologies and natural histories are not precisely defined, but they are thought to be associated with underlying conditions. In this report, we describe an extremely rare case of SRAD in a man who had a history of spontaneous SMAD.

## 2. Case Report

**Figure 1:**
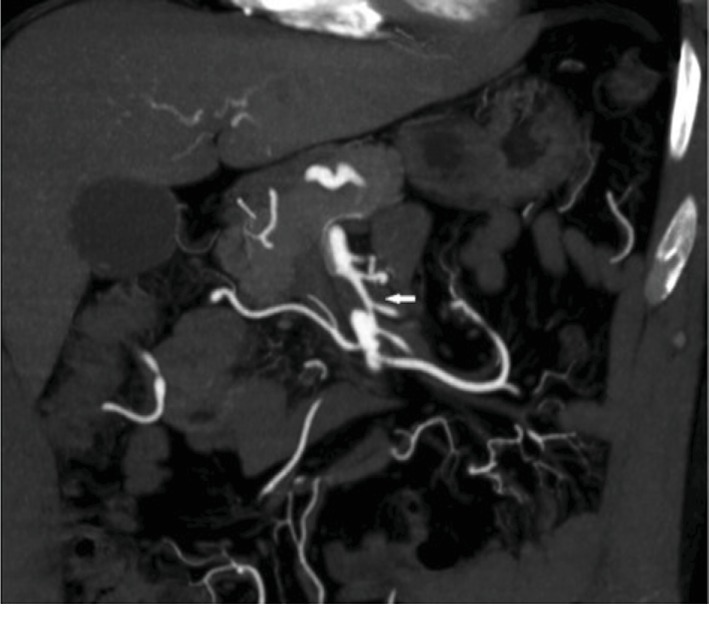
Dissection of the superior mesenteric artery (arrow).

A 41-year-old man presented to our hospital with a history of abdominal pain for the last 3 days. He had no past medical history. He did not have hypertension, hyperlipidemia, diabetes, or any collagen vascular disease. Contrast-enhanced computerized tomography (CT) showed SMAD without bowel ischemia (Figure 1). The patient was admitted and received 1 week of anticoagulant therapy. He was discharged from the hospital without complications and started taking 100 mg aspirin daily.

**Figure 2:**
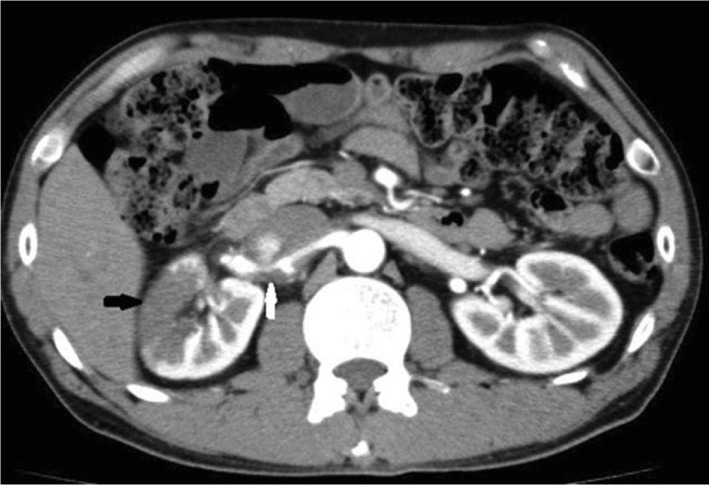
Right renal artery dissection (white arrow) and renal infarction (black arrow).

**Figure 3:**
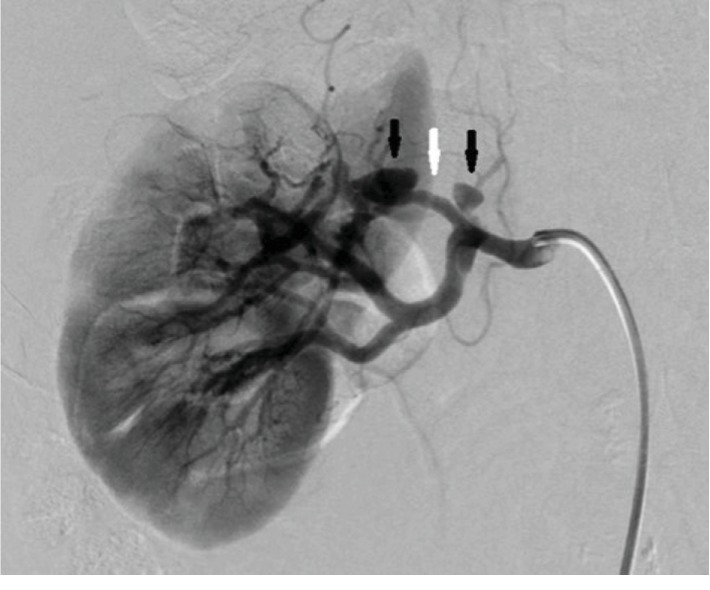
Aneurysms (black arrow) and narrowing (white arrow) due to dissection.

**Figure 4:**
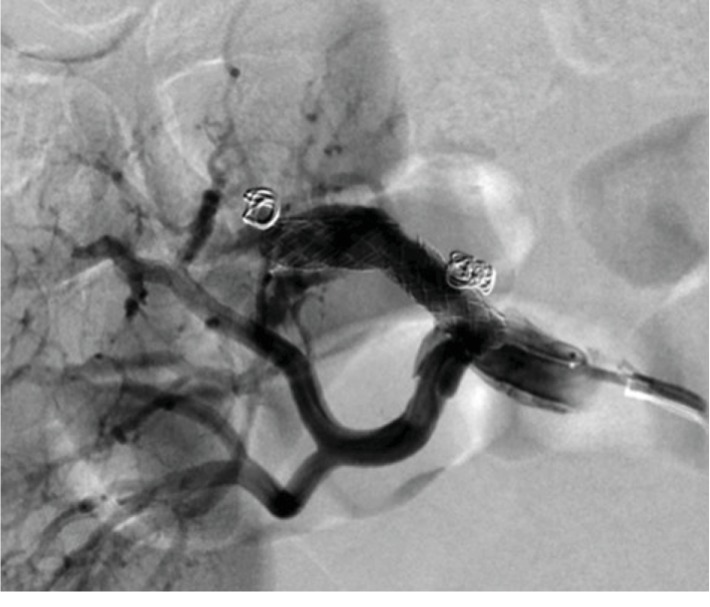
The false lumens are occluded by coiling, and the narrowing is diminished.

He was followed up once in 3 months, but 2 years later he was brought to our hospital with right-sided flank pain. His blood pressure was 142/96 mmHg, and his heart rate was 66 bpm. His abdomen was soft but not tender. Contrast-enhanced CT revealed right renal artery dissection with right renal infarction of the upper one-third portion (Figure 2). His serum creatinine level was not elevated. Renal angiography revealed two focal aneurysms and narrowing of the upper branch due to dissection (Figure 3). Two stents (Palmatz genesis 6.0 mm∗18 mm × 2) were deployed and the aneurysms were embolized by coiling (Target Detachable coils) in order to dilate the narrowing and obliterate the blood flow into aneurysms. We confirmed that the narrowing was diminished, that no blood flow entered the false lumens, and that no other branches were occluded (Figure 4).

Antiplatelet therapy with 100 mg aspirin daily was continued postoperatively and is to be continued indefinitely considering for his high risk for recurrence. His renal function did not decline, and contrast-enhanced CT performed 2 weeks after the intervention showed no advancement of renal infarction. He was discharged without complications.

He was followed up without symptom, and contrast-enhanced CT performed 6 months after discharge showed no advancement of both renal infarction and renal artery dissection. Genetic testing was performed but we could not find any abnormalities.

## 3. Discussion

SMAD is commonly associated with aortic dissection. Isolated spontaneous SMAD is a rare condition. Causes of spontaneous SMAD include atherosclerosis, fibromuscular dysplasia, segmental arterial mediolysis, connective tissue disorder, vasculitis like giant cell arteritis, Takayasu arteritis, and polyarteritis nodosa. It is accompanied by acute epigastric pain and may cause intestinal ischemia or intraabdominal rupture, but sometimes there are no symptoms [1]. There are three therapeutic approaches: conservative management, endovascular therapy, and surgical revascularization. Conservative management includes anticoagulants, antiplatelet drugs, blood pressure control, and pain control, but there is no consensus on the optimal strategy for conservative management. Surgical revascularization is performed in cases with bowel necrosis, aneurysm rupture, or failure of endovascular repair.

SRAD is also rare, and the most common presenting symptom is sudden-onset flank pain; it may cause uncontrolled blood pressure, renal infarction, and renal failure [2]. It may be caused by fibromuscular dysplasia, segmental arterial mediolysis, connective tissue disorder, or vasculitis. SRAD is difficult to diagnose because of its nonspecific symptoms. Renal ultrasound and Doppler have poor diagnostic sensitivity, so contrast-enhanced CT is the gold standard for diagnosis [3]. The treatment for SRAD has not been established, so treatment should be based on the patient's clinical presentation and the anatomical features of the dissection. Treatment includes supportive medical treatment, endovascular intervention, and surgery. Supportive therapy including control of hypertension and systemic anticoagulation has shown good outcomes, but there is still a risk of acute infarction or renovascular hypertension. Endovascular treatment is a good option because it can prevent both advancement of infarction and renovascular hypertension, and it has become safer as device technology has improved [4]. The potential complications of endovascular treatment are puncture site hematoma, development of dissection, renal artery thrombosis, or perforation. Contraindications for endovascular treatment is unstable hemodynamics. The intravascular ultrasound may be helpful for the evaluation of etiology [5].

A combination of spontaneous SMAD and SRAD is extremely rare [6, 7]. We cannot identify the etiology of this patient, but he may have an underlying condition like segmental arterial mediolysis, so regular follow-up with contrast-enhanced CT will be needed for such a case. Furthermore, if the patients have another abdominal or back pain, prompt workup like contrast-enhanced CT will be needed and proper interventions should be done immediately if necessary to prevent severe consequences like organ ischemia or hemorrhage.

## 4. Conclusion

A combination of spontaneous SMA dissection and SRAD is a very rare condition. Patients with isolated visceral artery dissection should be carefully followed up.

## References

[1] Tameo M. N., Dougherty M. J., Calligaro K. D. (2011). Spontaneous dissection with rupture of the superior mesenteric artery from segmental arterial mediolysis. *Journal of Vascular Surgery*.

[2] Afshinnia F., Sundaram B., Rao P., Stanley J., Bitzer M. (2013). Evaluation of characteristics, associations and clinical course of isolated spontaneous renal artery dissection. *Nephrology, Dialysis, Transplantation*.

[3] Gandhi S. P., Patel K., Pal B. C. (2015). Isolated spontaneous renal artery dissection presented with flank pain. *Case Reports in Radiology*.

[4] Tandon G., Sukhija R. (2012). Isolated spontaneous renal artery dissection: a case report and review. *International Journal of Angiology*.

[5] Peynircioğlu B., Pişkinkaya S., Özer Ç., Cil B., Yorgancioglu C., Arici M. (2009). Isolated spontaneous renal artery dissection: diagnosis and endovascular management. *Diagnostic and Interventional Radiology*.

[6] Srinivasan K. G., Srivdya S., Ushanandhini P., Ramprabanath S. (2009). Spontaneous isolated superior mesenteric artery dissection – report of two cases. *Journal of Radiology Case Reports*.

[7] Sugiura T., Imoto K., Uchida K. (2011). Fibromuscular dysplasia associated with simultaneous spontaneous dissection of four peripheral arteries in a 30-year-old man. *Annals of Vascular Surgery*.

